# Glycaemic outcomes in people living with diabetes under 65 and over 65 years old using an intermittently scanned continuous glucose monitoring system

**DOI:** 10.1177/20420188241269133

**Published:** 2024-08-20

**Authors:** Carol Wong, Anne De Bray, Naeem Ul Hassan, Ahmed Almohandes, Kyi Zin Thant, Sofia Gill, Dayna Gill, Hayley Forsdick, Alan J Sinclair, Muhammad Ali Karamat, Srikanth Bellary

**Affiliations:** Department of Diabetes and Endocrinology, Birmingham Heartlands Hospital, University Hospitals Birmingham NHS Foundation Trust, Birmingham, UK; Centre for Endocrinology, Diabetes and Metabolism, University of Birmingham, Birmingham, UK; Oxford Centre for Diabetes, University of Oxford, Oxford, UK; Department of Diabetes and Endocrinology, Birmingham Heartlands Hospital, University Hospitals Birmingham NHS Foundation Trust, Birmingham, UK; Department of Diabetes and Endocrinology, Birmingham Heartlands Hospital, University Hospitals Birmingham NHS Foundation Trust, Birmingham, UK; Department of Diabetes and Endocrinology, Birmingham Heartlands Hospital, University Hospitals Birmingham NHS Foundation Trust, Birmingham, UK; Birmingham Medical School, University of Birmingham, Birmingham, UK; Birmingham Medical School, University of Birmingham, Birmingham, UK; Department of Diabetes and Endocrinology, Birmingham Heartlands Hospital, University Hospitals Birmingham NHS Foundation Trust, Birmingham, UK; King’s College London, London, UK; Department of Diabetes and Endocrinology, Birmingham Heartlands Hospital, University Hospitals Birmingham NHS Foundation Trust, Birmingham, UK; Centre for Endocrinology, Diabetes and Metabolism, University of Birmingham, Birmingham, UK; Aston University, Aston Triangle, Birmingham B4 7ET, UK; University Hospitals Birmingham NHS Foundation Trust, Birmingham, UK

**Keywords:** continuous glucose monitoring, older adults, type 1 diabetes mellitus

## Abstract

**Objective:**

Intermittently scanned continuous glucose monitoring (isCGM) has revolutionised the care of people with diabetes but its uptake and benefits in older adults are not well known. We examined the impact of isCGM (Freestyle Libre, FSL) on glycaemic outcomes in younger (⩽65 years) and older adults (>65 years) with diabetes.

**Design and methods:**

In total, 2260 adult patients registered on the Libreview account at University Hospitals Birmingham NHS Foundation Trust, UK, were included. Inclusion criteria: all patients with type 1 and type 2 diabetes aged >18 years, use of isCGM >6 months, scanning at least 6 times/day. Demographics, diabetes history and glycaemic outcomes (time in range (TIR), time above range and time below range (TBR), estimated HbA1c, HbA1c at start and at end of study) were collected by accessing electronic patient records and Libreview. Outcomes were compared between age groups ⩽65 or >65 years old.

**Results:**

Most patients were of Caucasian ethnicity (⩽65 years 68%, >65 years 73%) and had type 1 diabetes. Mean duration of diabetes was 19.5 years (range 0–65 years) and 34.5 years (range 0–79 years) for ⩽65 and >65 years, respectively. Only a quarter of those ⩽65 years achieved (219/943; 23.2%) their age specific TIR target compared to 69% (78/113) of those >65 years cohort, while 70.1% (663/946) of ⩽65 years and 40.7% (46/113) of >65 years achieved their age-specific TBR target. When the less strict ⩽65 years TBR target was applied, 75% (85/113) of >65 years cohort achieved this.

**Conclusion:**

FSL use was associated with improved glycaemic outcomes across all age groups. Individualised targets may be needed to improve TBR in those aged >65 years.

## Introduction

Continuous glucose monitoring (CGM) was developed as a novel method of home glucose monitoring levels over 20 years ago.^1^ CGM involves measuring interstitial fluid glucose levels via a continually worn transdermal sensor and has been demonstrated to be acceptable to patients, safe and reduce time spent in hypoglycaemia^2,3^ when compared with traditional ‘finger prick’ capillary blood glucose (CBG) monitoring. In addition, CGM is less cumbersome than CBG monitoring, allows for easier review of glucose trends rather than solely the spot measurements of CBG monitoring and enables remote review of results by the clinical team.^4,5^ CGM can either be real-time (rt-CGM) or intermittently scanned (isCGM).^6^ We will use the Diabetes UK definitions^7^ and refer to rt-CGM as CGM only and isCGM is referred to as flash glucose monitoring. Flash glucose monitoring has been shown to improve^8^ or not worsen^9^ HbA1c, improve patient satisfaction,^8,10^ reduce hospitalisation^10–12^ and reduce work absenteeism.^10^ An additional benefit of isCGM is the reduction in time spent in hypoglycaemia for both people living with type 1^10,12,13^ and type 2^9,14^ diabetes.

Increasing duration of diabetes heightens the risk of severe hypoglycaemia^15^ and this risk is exaggerated in older adults with diabetes.^16,17^ Several age-related mechanisms including reduction in beta-adrenergic receptor function,^18^ impairment of counter-regulatory endocrine response^19^ and impaired glucagon response to hypoglycaemia are known to contribute to this.^20^ In addition, older adults are highly susceptible to events that can precipitate or worsen the effects of hypoglycaemia such as falls, dysrhythmias and cognitive impairment.^21,22^ CGM can play a key role in combating the hypoglycaemia unawareness from impaired counter-regulatory response, by predicting and reducing hypoglycaemia in this population, as demonstrated in the Wireless Innovation in Seniors with Diabetes Mellitus trial with a small but statistically significant reduction in time spent in hypoglycaemia following 6 months of CGM use compared with standard blood glucose monitoring.^23^ An additional benefit of CGM is that remote review of glucose management data can help clinicians direct management tailored to the patient, of particular utility when some older adults are limited in their ability to self-manage. With the bidirectional link between cognitive decline and hypoglycaemia risk well established,^24,25^ CGM has potential to disrupt that link by predicting hypoglycaemic episodes and facilitates the user or a carer to act to prevent the hypoglycaemic episode from occurring. Therefore, the emphasis of CGM use in older adults living with diabetes should be focused on reducing hypoglycaemia and avoiding excessive hyperglycaemia,^26,27^ rather than aiming for intensive glycaemic targets.

Although the raising of the target HbA1c range may reduce the risk of severe hypoglycaemia in some patients, there are some concerns with this. Increasing the HbA1c target does not eliminate the risk entirely with the risk severe hypoglycaemia having a u-shaped relationship to HbA1c, that is, risk is higher when HbA1c is very low or very high.^28,29^ In addition, increasing HbA1c target range may increase the risk of both macrovascular and microvascular complications associated with hyperglycaemia such as diabetic retinopathy, nephropathy and neuropathy.^28,29^

Therefore, adjusting target HbA1c range alone may be a blunt tool to prevent severe hypoglycaemia in older adults with diabetes while providing modest benefit for long-term complication risk reduction – this is where the additional glycaemic insights provided by CGM or flash glucose monitoring can be of benefit. Although there is some evidence that CGM reduces hypoglycaemia^23^ and glycaemic variability in older patients living with either type 1 or type 2 diabetes,^30^ there has not been extensive work examining the impact of isCGM in older adults living with diabetes. Considering that 16% of people over 65 years live with diabetes^31^ and those aged over 60 now constitute nearly half of adults with type 2 diabetes, focusing on optimising management for this cohort of people is of more importance than ever.

Improvements in sensor accuracy, usability, convenience and ultimately evidence-based improvement in glycaemia^32,33^ led NHS England to approve funding for isCGM (flash glucose monitoring device, Freestyle Libre, FSL) as an alternative to CBG testing in 2017.^34^ For children and young people living with type 1 diabetes, isCGM could be funded if they experienced frequent severe hypoglycaemia, impaired awareness of hypoglycaemia associated with adverse consequences or the inability to recognise or communicate about symptoms of hypoglycaemia.^34^ isCGM was funded for adults living with type 1 diabetes if they agreed to commit to using the device at least 70% of their time, had optimised insulin use and conventional blood glucose monitoring alongside one of several additional criteria.^33^ By 2019, NHS England estimates that approximately 3%s–5% of people living with diabetes in England had access to isCGM.^34^

The aim of this study was to review the current demographics of adults using isCGM and to review the provision of isCGM across different demographics (s age, social deprivation, ethnicity) and review glycaemic control of isCGM users in a large, teaching hospital Trust. In addition, this study aims to review how isCGM was being utilised by older patients and a snapshot of their glycaemic control in comparison with younger adults.

## Materials and methods

### Data collection

This study was a retrospective observational study of all adults registered on the Libreview account at University Hospitals Birmingham NHS Foundation Trust in June 2021 (*n* = 2260). The project was registered with the hospital Trust and approved by the governance team. At the time of this study, the only isCGM system funded by NHS England was FSL, manufactured by Abbott.^34^ Access to patient isCGM data is via the online database Libreview, in which NHS Trusts can curate their own patient lists. The patient or hospital team can adjust the individual patient Libreview account settings to a tailored target range.

Inclusion criteria were all patients over 18 years old registered on the Libreview account in June 2021 and who were scanning at least 6 times/day. To evaluate the outcomes across age groups, the cohort was divided into two groups based on age, ⩽65 or >65 years old, at the time of the commencement of isCGM. This division of age relates to the American Diabetes Association (ADA) guidelines^27^ where the glycaemic targets change for ⩽65 and >65 years old patients with diabetes.

**Figure 1.**
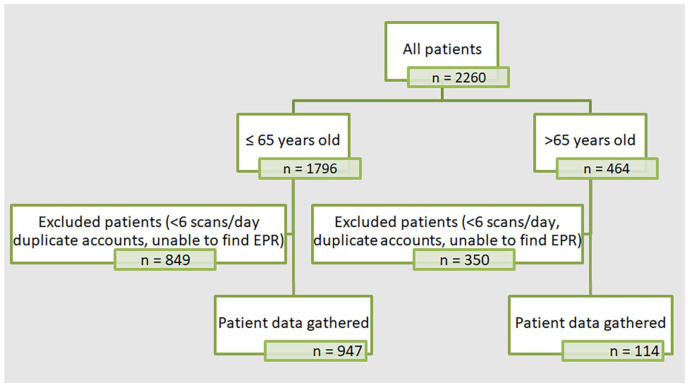
Methodology of patient selection. Data were collected from individual patient Libreview accounts and electronic patient records. Patient data gathered from Libreview included: average scans/views per day, average glucose, GMI (in % and mmol/mol), SD of GMI and, calculated over the preceding 3 months, percentage of TIR, TBR, TAR. EPR, electronic patient record; GMI, glucose management indicator; SD, standard deviation; TAR, time above range; TBR, time below range; TIR, time in range.

Patients accounts were excluded if (a) the patient was scanning fewer than 6 times/day, (b) if it was a duplicate account or (c) we were unable to find their electronic patient record (*n* = 1199 excluded; 849/1796 in ⩽65 years old cohort, 350/464 in >65 years old cohort) (Figure 1). The rationale for excluding those with scanning frequency below 6 times/day because optimal scanning frequency for local funding at the time was 6–8 scans/day^35^ and additionally, a lower scanning frequency would yield inaccurate related information, for example, the calculated time in range (TIR).

No patients were excluded on the basis of missing data; all patients were included for analysis in the categories for which information had been sourced, that is, for those without an indication for isCGM provision but had TIR data were included in TIR analysis.

Date of isCGM initiation was determined from the date of first regular scanning (>6 times/day) on Libreview or from local Diabetes Specialist Nurse clinic databases.

Information gathered from electronic patient records included: age, gender, ethnicity, postcode, type of diabetes, year of diabetes diagnosis, method of insulin delivery (pump/multiple daily injection – MDI), indication for isCGM, duration of isCGM usage, most recent HbA1c pre-FSL start and HbA1c at least 6 months post-FSL start. Data from isCGM were downloaded and for each patient, %TIR, % Time Below Range (TBR) and % Time Above Range (TAR) were recorded.

### Data analysis

Absolute and percentage change in HbA1c, and percentage of patients achieving their target TIR, TBR, TAR were calculated for both age groups. Age-specific targets for percentage of TIR, TBR and TAR readings were determined as per the 2019 ADA consensus guidelines for interpreting CGM data^27,34^; all patients >65 years were classed as ‘older/high risk’ patients. Due to the nature of the electronic patient records and data available for collection, we were unable to accurately determine if patients ⩽65 were ‘high risk’ patients, so all patients aged 65 or under were analysed against non ‘older/high risk’ patient targets.

Social deprivation data were calculated by postcode through the most recent West Midlands English Index of Multiple Deprivation (IMD) worksheet which allocated a deprivation decile. The lower the decile, the more deprived the postcode.^36^

Descriptive statistics (frequencies and cross tabulations, independent means test and Chi-square test) were used to describe the characteristics of the cohort. Categorical data are presented as % and continuous data are presented as mean (SD). The difference in pre and post libre HbA1c was analysed using independent means test. TIR, TAR and TBR were compared using Chi-square test. Data were analysed using SPSS 26 (IBM).

## Results

### Demographics and baseline characteristics

The cohort was divided into two specific age groups, ⩽65 years old (*n* = 947, mean 42.2, standard deviation 12.7) >65 years old (*n* = 113, mean 72 years, standard deviation 5.2). The number of patients (*n* = 2660) is reflective of real-life data and is the reason why there is a discrepancy in cohort size between ⩽65 or >65 years old. Across both groups, the gender distribution was similar; in the ⩽65 cohort, 52% were female versus 47% female in the over 65 cohort (Table 1).

**Table 1.** Summary of demographic and baseline characteristics.

Baseline characteristics	⩽65 years old, *N* = 947	>65 years old, *N* = 113	*p* value
Age (years)			
Mean (SD)	42.2 (12.7)	72.0 (5.2)	<0.001
Gender			0.390
Male (%)	48	53	
Female (%)	52	47	
Ethnicity, *N* (%)			
White European	630 (66.5)	82 (72.6)	0.169
South Asian	46 (4.9)	1 (0.9)	
Black	20 (2.1)	2 (1.8)	
Other/non-specified	236 (24.9)	26(23)	
IMD profile			
Mean IMD decile (standard deviation)	4.9 (3.1)	6.5 (3.2)	NS
Percentage of people in lowest 5 deciles (most deprived)	518 (58.1%)	43 (38%)	<0.001
Type of diabetes, *N* (%)			
Type 1	880 (93)	98 (87)	0.015
Type 2	23 (2)	7 (6)	
Type 3c	19 (2)	6 (5)	
Other^a^	25(3)	2 (2)	
Insulin regimen, *N* (%)			
MDI	655 (69)	86 (75)	0.169
CSII	286 (30)	27 (24)	
Unclear	6 (0.6)	1 (0.9)	
Diabetes duration (years) Mean (SD)	19.5 (13.8)	34.5 (17.4)	<0.01

All diagnoses of type 3c diabetes were due to pancreatitis. Further breakdown of ethnicity is provided in Supplemental Material.

a Other group includes: MODY, CFRD, LADA, CPI-mediated, MDI and CSII.

CFRD, cystic fibrosis related diabetes; CPI-mediated, checkpoint inhibitor-mediated; CSII, continuous subcutaneous insulin infusion; IMD, index of multiple deprivation; LADA, latent autoimmune diabetes in adults; MDI, multiple daily injections; MODY, maturity onset diabetes of the young.

Both cohorts had a similar spread of ethnic backgrounds with white Europeans constituting the largest group (723/1061; 68.1%) followed by South Asian (45/1061; 4.2%). Ethnicity for 239 (22.5%) of the total patient cohort were not specified, 217/947 (22.9%) nonspecified in the ⩽65 cohort and 22/114 (19%) not specified in the over 65 cohort.

Ethnicity across the West Midlands is diverse, and the data in the study were comparable to the cohort of people living with type 1 diabetes in Birmingham and Solihull; within the NHS Birmingham and Solihull CCG cohort, according to the National Diabetes Audit 2021/2022,^16^ 67.3% are white, 27.7% are minority ethnic origin and 5% unknown/not stated.

General characteristics of the cohorts at the time of data collection are summarised in Table 1.

A greater proportion of patients in the younger cohort (58%) were living in deprived postcodes compared to those aged >65 years (38%). The majority of patients across the two age cohorts had type 1 diabetes, with a higher proportion in the younger cohort 880/935 (93%), compared to the older cohort 97/114 type 1 (85%).

Most patients in both age cohorts received insulin via MDI therapy; ⩽65 cohort 655/947 (69%), >65, 86/114 (75%) compared to pump therapy; ⩽65 cohort 286/947 (30%), over 65 27/114 (24%). The insulin regime for MDI patients was not further detailed in the data collection. As would be expected, diabetes duration varied between the groups with those aged >65 years, having a mean duration of 34.5 years (standard deviation 17.4) compared to mean duration of 19.5 years (standard deviation 13.8) in the ⩽65 group.

#### Indication for FSL

The most common indication for isCGM in the younger cohort was intensive monitoring whereas in older adults, the intensive monitoring and hypoglycaemia were both common (Table 2).

**Table 2.** Frequency of patients with different indications for isCGM.

Indications for isCGM	⩽65 years old, *n* = 947	>65 years old, *n* = 114
Intensive monitoring	412 (43.5%)	31 (27.2%)
Hypoglycaemia	83 (8.8%)	25 (21.9%)
MDT decision/psychosocial/occupational	145 (15.3%)	8 (7.0%)
Self-funding	108 (11.4%)	5 (3.5%)
More than one indication	17 (1.8%)	8 (7.0%)
Pregnancy	18 (1.9%)	0 (0.0%)
Dialysis	3 (0.3%)	0 (0.0%)
Disability	4 (0.4%)	2 (1.8%)
Unclear	192 (20%)	51 (44.7%)

For some patients, there was more than one indication, so there are more indications than the total number of patients.

Mean duration of usage of isCGM was similar in both age groups (16 vs 18 months) in younger and older cohorts, respectively. The median number of scans/views per day, however, was also similar for both cohorts (Table 3).

**Table 3.** Duration and usage of FSL in both cohorts and glycaemic outcomes.

	⩽65 years old	>65 years old	*p* value
Duration of FSL usage (mean, standard deviation)	1 year 4 months (9 months)	1 year 6 months (10 months)	0.77
Number of scans/views per day (median, range)	11 (6–84)*	11 (6–36)*	0.409
Glycaemic outcomes Mean (SD)			
HbA1c pre-FSL (mmol/mol)	68.7 (17.4)	65.7 (11.7)	0.109
HbA1c ⩾6 months post-FSL (mmol/mol)	61.9 (14.1)	63.5 (11.2)	0.319
Mean change in HbA1c post-FSL (mmol/mol)	−6.8 (15.5)	−1.9 (7.9)	0.004
Average glucose (mmol/L)	9.6 (2.1)	9.5 (1.8)	0.553
Average GMI (mmol/mol)	58.0 (9.9)	57.4 (8.8)	
Time in range			
Number of patients with TIR >70%	219/943 (23.2%)	26/113 (23.0%)	0.959
Number of patients with TIR >50%	594/943 (63.0%)	78/113 (69.0%)	0.207
Time below range			
Number of patients with TBR <1%	351/946 (37.1%)	46/113 (40.7%)	0.454
Number of patients with TBR <4%	663/946 (70.1%)	85/113 (75.2%)	0.257
Time above range			
Number of patients with TAR <10%	53/937 (5.7%)	5/111 (4.5%)	0.616
Number of patients with TAR <25%	226/937 (24.1%)	27/111 (24.3%)	0.962

* For <65 years cohort, the second highest scanning frequency was 60 times/day. For >65 years cohort, the second highest scanning frequency was 36 times/day. Further review of the patients with very high scanning frequencies has revealed that most of these patients now scan less frequently but aim to keep their glycaemic control very tight. One patient continues to scan very often due to concerns about hypoglycaemia with exercise. Hba1c and glucose comparisons were undertaken using independent means test. TIR, TAR and TBR were compared using Chi-square test. *p* values shown are two-sided significance.

FSL, Freestyle Libre; GMI, glucose management indicator; SD, standard deviation; TAR, time above range; TBR, time below range; TIR, time in range.

#### Glycaemic outcomes

At the time of commencing isCGM monitoring, the >65 cohort had a lower starting HbA1c (mean HbA1c 65.7 mmol/mol) compared to the younger cohort (mean HbA1c 68.7 mmol/mol; Table 3). After at least 6 months of isCGM usage, the HbA1c in both cohorts decreased and was more pronounced in the ⩽65 cohort compared to the >65 cohort (mean change in Hba1c: 6.8 vs 1.9 mmol/mol, respectively). HbA1c was not routinely monitored post-FSL initiation in 19% of <65 years cohort and 15% of >65 years cohort, respectively. Average glucose and glucose management index (GMI) was similar between the two cohorts (Table 3).

##### Time in range, time below range and time above range

Results for TIR, TBR and TAR were taken as a snapshot at the time of data collection and were calculated from the preceding 3 months of measurements (Table 3).

In both groups, the proportion of patients achieving the TIR target >70% and TIR target >50% were similar (Table 3). However, when reviewed in the context of age-specific targets, 23.2% (219/943) of our ⩽65 years population achieved the age-specific TIR target of >70% (14) compared to 69% (78/113) of our >65 years cohort who achieved the age-specific target of >50% TIR.^27^

Patients ⩽65 years should aim for TBR <4% (27); 70.1% (663/946) of patients aged <65 years achieved the TBR target of <4% compared to 75.2% of those aged >65 years. When applying stricter criteria for TBR in older adults, 40.7% (46/113) achieved the target of TBR <1%.

Patients ⩽65 years should aim for TAR <25% (14); 24.1% (226/937) and 24.3% (27/111) of ⩽65 and >65 years, respectively, achieved the TAR target of <25%. However, a stricter target of TAR < 10% was achieved in only 4.5% (5/111) of the >65 cohort (Table 3).

It should be noted that the other than the mean change in HbA1c post-FSL, the *p* values do not hold statistical power and so there are limited conclusions to draw from these percentages. However, on a service evaluation level, it is a helpful insight as to where glycaemic control could be improved.

## Discussion

Our data, derived from a large cohort of patients living with diabetes, show encouraging trends in uptake and certain glycaemic outcomes across all age groups within the early years following the introduction of isCGM in England. Furthermore, to the best of our knowledge, this is the first real-world data comparing the use and impact of isCGM in younger and older adults.

Although the broader demographic characteristics (gender, ethnicity, mode of insulin delivery, type of diabetes) were similar across both age groups, there were some key differences. Social deprivation was higher in ⩽65 years patients, which may reflect the cumulative opportunity for social mobility in older patients. In addition, this difference in deprivation scores may be explained by our catchment area which includes an area popular with an older and more affluent population.

With the updated National Instituite of Clinical Excellence (NICE) guidance expanding funding eligibility to offer flash glucose monitoring to all patients with type 1 diabetes and allow consideration for certain patients taking insulin for type 2 diabetes,^13^ we expect the uptake of isCGM to increase, particularly in older adults with type 2 diabetes. Given that type 2 diabetes is more prevalent in people of black and south Asian ethnicities,^37^ there is a need to ensure equity of access to these technology across all ethnic groups.

Ten percent of the individuals in our study were aged >65 years. This largely reflects the proportion of those with type 1 diabetes in this age group and shows that the access to isCGM is uniform regardless of age. Typically, this cohort of patients are prone to hypoglycaemia due to the longer duration of diabetes and age-related changes. Fitting with the need to reduce risk of hypoglycaemia, the most common indications for isCGM initiation in our >65 cohort were intensive monitoring and reducing risk of hypoglycaemia. This was different to that in the <65 year cohort where intensive control was the most common indication. This may explain the difference in HbA1c from baseline which was more marked in the ⩽65 cohort despite a similar baseline HbA1c to the >65 cohort. Many patients did not have an indication for isCGM recorded, this is mostly explained by variation in clinician documentation. The change in funding criteria policy^26^ now removes the requirement for any such indication for people living with type 1 diabetes and the policy was released after the data collection began so would not have affected these missing data.

A reasonable proportion of patients in both cohorts were able to spend >60% of their time within their target glucose range although in our >65 cohort, less than 40% were able to spend <1% of their TBR (Table 3). When we applied the lower risk target for TBR (<4% time), the majority of >65 patients were achieving this. The TBR < 4% statistics were similar in both groups but in practice, we should aim for a higher proportion of older adults with TBR < 1%. The fact that nearly 60% older adults are not achieving this would indicate a number of older adults are probably still overtreated. The data collected on TBR were collected as a snapshot at one point in time so we are unable to infer the impact of isCGM on TBR over time or against a control group not using isCGM. However, it highlights the need to confirm knowledge of this stricter ADA target for older adults’ TBR in staff and patients. It is also important to emphasise that older adults comprise a heterogeneous group with varying levels of risk of hypoglycaemia and targets for TBR may need to be individualised as appropriate.

It would have been interesting to perform subgroup analysis of isCGM usage and glycaemic outcomes within the >65 cohort at different age groups with increasing frailty^35^ (e.g. >75 or >80 years), in association with frailty indicators or in relation to duration of diabetes. This is important considering frail older adults are at a greater risk of hypoglycaemia and CGM may help reduce this risk by recognising trends towards hypoglycaemia. It is also well recognized that hypoglycaemia and hypo unawareness increases with diabetes duration.^15^ However, this was limited by sample size (*n* = 20 for >75 years patients), study design and the absence of frailty indicators. Given that the latest NICE funding CGM criteria is likely to widen access of the technology to larger numbers of older adults, our next steps are to repeat the study with assessing frailty in mind.

## Limitations

Data were captured from electronic patient records based in secondary care. Therefore, the interpretation of some metrics may not be representative as some missing data (such as HbA1c) may be due to tests being carried out in primary care practices that do not link results with our Trust. In addition, the loss of HbA1c measurement post-isCGM initiation could have been due to the disrupted service provision during the coronavirus pandemic and clinician-led acceptability of utilising TIR, glycaemic variability and GMI as markers of glycaemic control in lieu.^38^ Our glycaemic outcomes were a snapshot of current glycaemic control. With TIR becoming a commonly used measure of glycaemic control,^24^ it would be valuable to review the trend of TIR from initiation to present day in comparison to HbA1c. Given the observational nature of this study and the lack of a control arm, our findings would require careful interpretation and need confirmation in future randomised studies. In addition, although we separated our cohorts into those ⩽65 years and those >65 years to reflect the ADA guidance we were applying, we appreciate that there are significant physiological differences between different extremes of each age range which may influence glycaemic control.

The snapshot observational nature of the service evaluation meant that there were no control groups without iSCGM use to compare to. Further research studies with designed study aims could answer questions relating to pathologies associated with diabetes and their association with the use of CGM. Data were not collected relating to these pathologies.

Since the start of this project, technology and guidelines in the United Kingdom for diabetes care has advanced. Initially isCGM was limited to specific indications but while completing this study, most patients in the United Kingdom with type 1 diabetes have access to isCGM now. As such, it would be difficult to compare any outcomes with future cohorts without isCGM compared to those with isCGM.

Longer diabetes duration has been shown to be associated with increased risk of hypoglycaemic episodes. In our study, as expected, older adults had a longer duration of diabetes which was significant compared to those under 65 years. We did not specifically look at the interaction between duration and TBR as in routine practice these results are accepted alongside the duration of diabetes and therefore unable to comment if duration affected TBR values in older adults.

## Conclusion

Noninvasive glucose monitoring has revolutionised the management of type 1 and type 2 diabetes offering greater convenience to patients and more importantly encouraging self-management and improving glycaemic outcomes. Our data show that these benefits are extended to all age groups, particularly in older adults and those with long-standing diabetes who are at greater risk of hypoglycaemia.

## Supplemental Material

sj-docx-1-tae-10.1177_20420188241269133 – Supplemental material for Glycaemic outcomes in people living with diabetes under 65 and over 65 years old using an intermittently scanned continuous glucose monitoring system Supplemental material, sj-docx-1-tae-10.1177_20420188241269133 for Glycaemic outcomes in people living with diabetes under 65 and over 65 years old using an intermittently scanned continuous glucose monitoring system by Carol Wong, Anne De Bray, Naeem Ul Hassan, Ahmed Almohandes, Kyi Zin Thant, Sofia Gill, Dayna Gill, Hayley Forsdick, Alan J Sinclair, Muhammad Ali Karamat and Srikanth Bellary in Therapeutic Advances in Endocrinology and Metabolism

## Declarations

**Ethics approval and consent to participate:** This project was submitted as a clinical audit with the local hospital trust and had approval granted from the governance team. This project did not include new treatment or practice beyond routine clinical management, random allocation or use of control groups. According to the UKRI/HRA decision tool, this project did not require ethics approval. To comply with the Caldicott Principles (1997) and Data Protection Act (1998) and to ensure patient anonymity, personal details including hospital ID number, name, address and date of birth were not recorded and were coded using a unique identifier. Data were kept securely, accessed only via NHS trust computers and email. Section 60 of the Health and Social Care Act 2001 provides an interim power to ensure that patient identifiable information, needed to support a range of important work such as clinical audit, record validation and research can be used without the consent of patients.

**Consent for publication:** There were no patient images, data reported in this service evaluation were in keeping with Section 60 of the Health and Social Care Act 2001.

**Data access and responsibility: Guarantor statement:** SB and MAK are the guarantors of this work and, as such, had full access to all the data in the study and takes responsibility for the integrity of the data and the accuracy of the data analysis.

**Availability of data and materials:** None.
