# Supplementary material for: Glycaemic outcomes in people living with diabetes under 65 and over 65 years old using an intermittently scanned continuous glucose monitoring system
Source: Ther Adv Endocrinol Metab. 2024 Aug 20;15:20420188241269133. doi: 10.1177/20420188241269133 (PMC11337186; doi:10.1177/20420188241269133)
Supplement: sj-docx-1-tae-10.1177_20420188241269133 – Supplemental material for Glycaemic outcomes in people living with diabetes under 65 and over 65 years old using an intermittently scanned continuous glucose monitoring system [file sj-docx-1-tae-10.1177_20420188241269133.docx]

# Supplementary material 1

Further detail of ethnicity within cohorts.

| **Characteristic** | | | |
| --- | --- | --- | --- |
|  | Overall | ≤65 years old | >65 years old |
| **Ethnicity** | | | |
| **n** | 1061 | 947 | 114 |
| South Asian | 45 (4.2%) | 43 (4.5%) | 2 (1.8%) |
| *Asian/British* | 6 | 6 | 0 |
| *Asian/British/Bangladeshi* | 1 | 1 | 0 |
| *Asian/British/Indian* | 24 | 22 | 2 |
| *Asian/British/Other* | 8 | 8 | 0 |
| *Asian/British/Pakistani* | 6 | 6 | 0 |
| Black | 22 (2.1%) | 20 (2.1%) | 2 (1.8%) |
| *Black/British* | 4 | 4 | 0 |
| *Black/British/African* | 3 | 3 | 0 |
| *Black/British/Caribbean* | 10 | 9 | 1 |
| *Black/British/Other* | 3 | 3 | 0 |
| *Caribbean* | 2 | 1 | 1 |
| Chinese | 2 (0.1%) | 2 (0.2%) | 0 (0%) |
| Mixed | 13 (1.2%) | 12 (1.3%) | 1 (0.9%) |
| *Mixed/other* | 3 | 3 | 1 |
| *Mixed/White/Asian* | 3 | 3 | 0 |
| *Mixed/White/Black/African* | 2 | 2 | 0 |
| *Mixed/White/Black/Caribbean* | 4 | 4 | 0 |
| White | 723 (68.1%) | 648 (68.4%) | 84 (73.7%) |
| *White/Irish* | 8 | 7 | 1 |
| *White/British* | 705 | 623 | 82 |
| *White/Other* | 19 | 18 | 1 |
| Other | 8 (0.8%) | 5 (0.5%) | 3 (2.6%) |
| N/A | 239 (22.5%) | 217 (22.9%) | 22 (19%) |
